# A hierarchical dynamic model used for investigating feed efficiency and its relationship with hepatic gene expression in APOE*3‐Leiden.CETP mice

**DOI:** 10.14814/phy2.14832

**Published:** 2021-05-01

**Authors:** Xiang Zhang, Yared Paalvast, Yanan Wang, Patrick C. N. Rensen, Albert K. Groen

**Affiliations:** ^1^ Department of Experimental Vascular Medicine Amsterdam University Medical Center University of Amsterdam Amsterdam The Netherlands; ^2^ Human and Animal Physiology Wageningen University Wageningen The Netherlands; ^3^ Theoretical Biology and Bioinformatics Utrecht University Utrecht The Netherlands; ^4^ Department of Pediatrics University Medical Center Groningen Groningen The Netherlands; ^5^ Department of Medicine Division of Endocrinology Leiden University Medical Center Leiden The Netherlands; ^6^ Einthoven Laboratory for Experimental Vascular Medicine Leiden University Medical Center Leiden The Netherlands

**Keywords:** feed efficiency, liver, metabolic syndrome, RNA‐Seq, transgenic mice

## Abstract

**Background:**

Feed efficiency (FE) is an important trait for livestock and humans. While the livestock industry focuses on increasing FE, in the current obesogenic society it is more of interest to decrease FE. Hence, understanding mechanisms involved in the regulation of FE and particularly how it can be decreased would help tremendously in counteracting the obesity pandemic. However, it is difficult to accurately measure or calculate FE in humans. In this study, we aimed to address this challenge by developing a hierarchical dynamic model based on humanized mouse data.

**Methods:**

We analyzed existing experimental data derived from 105 APOE*3‐Leiden.CETP (E3L.CETP) mice fed a high‐fat high‐cholesterol (HFHC) diet for 1 (N = 20), 2 (N = 19), 3 (N = 20), and 6 (N = 46) month. We developed an ordinary differential equation (ODE) based model to estimate the FE based on the longitudinal data of body weight and food intake. Since the liver plays an important role in maintaining metabolic homeostasis, we evaluated associations between FE and hepatic gene expression levels. Depending on the feeding duration, we observed different relationships between FE and hepatic gene expression levels.

**Results:**

After 1‐month feeding of HFHC diet, we observed that FE was associated with vitamin A metabolism, arachidonic acid metabolism, and the PPAR signaling pathway. After 3‐ and 6‐month feeding of HFHC diet, we observed that FE was associated most strongly with expression levels of *Spink1* and *H19*, genes involved in cell proliferation and glucose metabolism, respectively.

**Conclusions:**

In conclusion, our analysis suggests that various biological processes such as vitamin A metabolism, hepatic response to inflammation, and cell proliferation associate with FE at different stages of diet‐induced obesity.

## Graphical abstract

Feed efficiency changes over time and varies between animals.

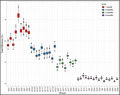

## 1 BACKGROUND

Obesity in humans is a global health problem and is primarily caused by excessive caloric intake that accompanies a sedentary lifestyle, a so‐called positive energy balance. However, despite the fact that most people in the developed world live in an obesogenic environment, some become obese and others do not. Understanding the mechanism underlying inter‐individual variations in progressing weight gain will provide us with novel therapeutic targets to counteract the obesity pandemic.

In livestock, the propensity toward weight gain has been quantified in the form of feed efficiency (FE), which is defined as the ratio of calories consumed divided by body weight gain over a specific time interval. As an important economic trait, FE is used for selecting the most feed‐efficient animals in order to reduce food consumption for the same amount of meat or dairy production. Since the liver plays a central role in partitioning and metabolizing nutrients, genome‐wide transcriptomic analyses have been performed in pigs and cows to investigate metabolic processes that are related to FE. It has been shown that vitamin A metabolism, lipid metabolism, and hepatic response to inflammation are associated with FE in these animals (Alexandre et al., 2015; Horodyska et al., 2019; Mukiibi et al., 2018; Ramayo‐Caldas et al., 2018; Zhao et al., 2016). Whether these processes are also related to the propensity toward weight gain in humans is not known.

To be able to calculate FE accurately, food intake has to be monitored continuously and housing conditions should be kept constant. Obviously, this is difficult in humans. In this study, we therefore used a humanized mouse model, the APOE*3‐Leiden.CETP (E3L.CETP) mouse (Westerterp et al., 2006) as a proxy to study FE and its regulation in humans. The E3L.CETP mouse is a promising translational model for studying diet‐induced obesity and metabolic syndrome (Hoek et al., 2014). It has a humanized lipoprotein metabolism and can develop metabolic syndrome within several months upon feeding a high‐fat high‐cholesterol (HFHC) diet (Paalvast et al., 2017; Rozendaal et al., 2018). Interestingly, despite an identical genetic background and housing condition, HFHC diet feeding E3L.CETP mice showed heterogeneous body weight gain after a similar amount of food consumption and well‐controlled housing conditions (Nasias et al., 2019; Tarasco et al., 2018).

Here we used existing experimental data derived from 105 E3L.CETP mice fed a HFHC diet for 1 (N = 20), 2 (N = 19), 3 (N = 20) and 6 (N = 46) month (Paalvast et al., 2017). To gain insight in FE regulation, we analyzed hepatic transcriptomes of 43 E3L.CETP mice (1 month N = 9; 2 months N = 10; 3 months N = 8; 6 months N = 16). Since FE is the outcome of a complex balance between calorie intake, energy expenditure, and calorie excretion, FE changes over time (Rozendaal et al., 2019). In order to estimate the FE at the time point corresponding to the transcriptomic analyses, we developed an ordinary differential equation (ODE) model based on the longitudinal data of food intake and body weight. We then assessed the associations between FE and gene expression levels in the liver.

## 2 METHODS

### 2.1 Animals, diet, and housing

#### 2.1.1 Paalvast study

In a first study, male E3L.CETP mice (C57BL/6 J background; N = 105, at the age of 4 months) were housed individually in a light ((lights on at 7:00 AM,lights off at 7:00 PM) and temperature (21°C) controlled facility, and were fed a synthetic HFHC diet (D12492, Research Diets) containing 60% fat and 0.25% cholesterol. The energy density of the HFHC diet was 5.21 kcal/g, with fat, carbohydrate, and protein providing 60%, 20%, and 20% energy. Among the 105 E3L.CETP mice, 20, 19, 20, and 46 mice were fed *ad libitum* for 1, 2, 3, and 6 months, respectively. Details of the experimental setup were previously described (Paalvast et al., 2017).

#### 2.1.2 Rozendaal study

In a second study, male E3L.CETP mice (N = 8, at the age of 11 weeks) were co‐housed in a light and temperature‐controlled facility, and fed the same synthetic HFHC diet (D12492, Research Diets). These E3L.CETP mice were fed *ad libitum* for 3 months. Experimental details were previously described in (Rozendaal et al., 2018;14:e^1006145^.).

Both studies were approved by the Ethics Committees for Animal Experiment of the University of Groningen and Leiden University. All methods were carried out in accordance with relevant guidelines and regulations. All mice were obtained from TNO, Innovation for Life, The Hague, The Netherlands. Anesthetic and euthanasia methods were not applicable in this study. All methods were carried out in accordance with ARRIVE guidelines.

### 2.2 Computational model

Our computational model is composed of a system of coupled, nonlinear ordinary differential equations (ODEs).

$$\frac{\mathrm{dF}}{\mathrm{dt}}=\text{FI}$$

$$\frac{\mathrm{dW}}{\mathrm{dt}}={\text{FE}}_{\max}\left(1‐\frac{W\left(t\right)}{W_{\max}}\right)\text{FI}$$

This model contains two state variables, namely cumulative food consumption (*F*) and body weight (*W*). Meanwhile, there are three system parameters, including FI (food intake, g/day), FE*_max_* (maximum feed efficiency), and *W_max_* (maximum body weight, g).

In order to address heterogeneity across mice, we allowed individual mice to have their own FI and incorporated the ODE model in a hierarchical model. We estimated the model parameters by applying Markov Chain Monte Carlo implemented in the program, Stan.

#### 2.2.1 Prior distributions for system parameters

Based on a previous study consisting of the same animal model and diet (Tarasco et al., 2018), we used a normal distribution with a mean 2.5 g/day and a standard deviation 0.1 g/day as the prior distribution to describe the mean daily food intake of E3L.CETP mice. Based on that study, we used an exponential distribution with the rate parameter 0.02 as the prior distribution for the maximum body weight. Since the maximum feed efficiency must be between 0 and 1, we used a non‐informative *Beta*(2,8) as the prior distribution. For the remaining parameters, we applied the non‐informative prior distributions.

#### 2.2.2 Initial values

In this study, day 0 refers to the day when the mice received the HFHC diet. Hence, cumulative food consumption at day 0 (*F*(*t* = 0)) was set as 0. Body weight measurements at day 0 were used as initial values.

#### 2.2.3 Implementation details

The mathematical model and multi‐level model were implemented in Stan (2.19.1) (Carpenter et al., 2017). The ordinary differential equations were solved by running the function integrate_ode_bdf implemented in Stan. We fitted the model by running Hamiltonian Markov Chain Monte Carlo in the program Stan (version 2.19.1) (Carpenter et al., 2017). We ran four Markov chains with 2000 iterations in each chain. Results were presented with the posterior mean with a 95% credible interval. The code can be found at https://github.com/XiangZhangSC/mice_to_human

### 2.3 Model validation

To validate our dynamic model, we applied it to a study with the same mice fed the same diet performed in a different laboratory. We simulated 8 E3L.CETP mice were described in a previously published study (Rozendaal et al., 2018). For each mouse, we ran 1000 simulations. In each simulation, we used body weight at day 0 as the initial value of body weight, and we set the initial value of food consumption as 0. The simulated body weight values were represented by the mean together with a 95% credible interval. The actual body weight measurements for each animal were imposed in the same plot.

### 2.4 RNA sequencing analysis

In total, 43 HFHC‐feeding E3L.CETP mice (1‐month group: N = 9, 2‐month group: N = 10, 3‐month group: N = 8, 6‐month group: N = 16) were randomly selected from each feeding group. Their liver tissues were prepared for RNA sequencing analysis. RNA of the liver tissue was isolated by using a commercially available kit (RNeasy, Qiagen). Quality control of RNA samples was performed by capillary electrophoresis using LabChip GX (Perkin Elmer). Libraries were constructed by using the 3’QuantSeq sample preparation kits (Lexogen). The obtained cDNA fragment libraries were sequenced on an Illumina HiSeq2500. The obtained reads were aligned to the mouse reference genome (ensembl release 82) by running (hisat). The gene level quantification was performed by running HTSeq‐count (Anders et al., 2015). More details were described in a previous study (Paalvast et al., 2017).

### 2.5 Hepatic triglycerides

Liver lipids were extracted using the Bligh and Dyer procedure (Bligh & Dyer, 1959) and redissolved in 2% Triton‐X100. Triglycerides were measured using a commercially available kit (Roche).

### 2.6 Endogenous glucose production

Endogenous glucose production was determined as described (Paalvast et al., 2017). In brief, food was removed at 9 AM and the experiment started at 1 PM. At 1 PM, animals received 0.1 mg/g [6,6]‐D2‐glucose intraperitoneally at a concentration of 30 mg/mL. Immediately before, and 10, 20, 30, 40, 50, 60, 75, and 90 min after the intraperitoneal injection, blood glucose was measured with an Accu‐Check glucose meter, and a small amount of blood was collected on a filter paper. Glucose was then extracted from the dried blood with a water/ethanol mixture. After the solution was evaporated under nitrogen flow at 60°C, the residue was derivatized to glucose penta‐acetate by adding 100 *µ*L pyridine and 200 *µ*L acetic anhydride to the extracted glucose and heating at 60°C for 30 min. After evaporation under nitrogen flow, the residue was then dissolved in 200 *µ*L ethyl acetate for analysis by GC–MS. GC–MS was performed with a Zebron ZB‐1701 30 m × 250 *µ*m × 0.25 *µ*m (Phenomenex) column under positive chemical ionization with ammonia ions monitored at m/z 408–412 (m_0_–m_4_). After corrections for natural abundances, the kinetics of the enrichment of glucose with D2‐glucose were then used to estimate endogenous glucose production.

### 2.7 Statistical analysis

To gain insights in biological processes that are related to feed efficiency, we evaluated associations between feed efficiency and hepatic gene expression levels. We focused on the 43 E3L.CETP mice that had RNA sequencing data (Paalvast et al., 2017). The response variable *y* is a matrix with 22897 rows and 43 columns. Each row of *y* represents a gene and each column of *y* represents a mouse. Every entry in this matrix represents the frequency that a gene *i* was observed in the mouse *j*. We focused on genes that have nonzero count in at least 40 samples. In total, we assessed associations of feed efficiency with 11390 genes. Since there were four cohorts of mice, we performed the association analysis within each group. The predictor variable *x* is a matrix has 1000 columns, and 9, 10, 8, or 16 rows depending on the mouse cohort. Every row of *x* represents a mouse and every column of *x* represents a simulation. Since the gene expression data were derived from the liver on the day that the animal was sacrificed, we calculated the feed efficiency on the same day by solving the ODE system. Since feed efficiency is unobserved, we calculated the feed efficiency 1000 times for every mouse in order to take into account the uncertainty. In the next step, we ran negative binomial regression analyses between liver gene expression and feed efficiency adjusting for the batch effects (different day and chemical reactant for RNA isolation) by running the function glm.fit implemented in the R package edgeR (McCarthy et al., 2012; Robinson et al., 2010). Within each iteration, we first calculated the *P* value based on the likelihood ratio test implemented in the glmLRT function and then corrected for the multiple testing by calculating the false discovery rate (FDR). If a gene had FDR below 0.05, that gene was considered as differentially expressed. Within each iteration, we also performed KEGG (Kyoto Encyclopedia of Genes and Genomes) pathway analysis by running the kegga function in the edgeR package (Robinson et al., 2010). The hypergeometric test was used to calculate the *P* values. Regarding the issue of multiple testing, we calculated the FDR for each pathway. After 1000 iterations, we counted how many times an association between a gene (or a pathway) and the feed efficiency had a false discovery rate below 0.05.

We assessed the relationship between feed efficiency and hepatic triglycerides as well as hepatic glucose production rate by performing Spearman correlation analysis. We focused on the E3L.CETP mice that were fed by HFHC diet for 1 month. There were 10 1‐month feeding E3L.CETP mice having measurements of hepatic triglycerides. There were 14 6‐month feeding E3L.CETP mice having measurements of hepatic glucose production rate. We performed the Spearman correlation analysis 1000 times and counted how many times the corresponding *P* value is below 0.05.

## 3 RESULTS

### 3.1 Dynamic model predicted body weight changes in E3L.CETP mice fed a HFHC diet

**FIGURE 1:**
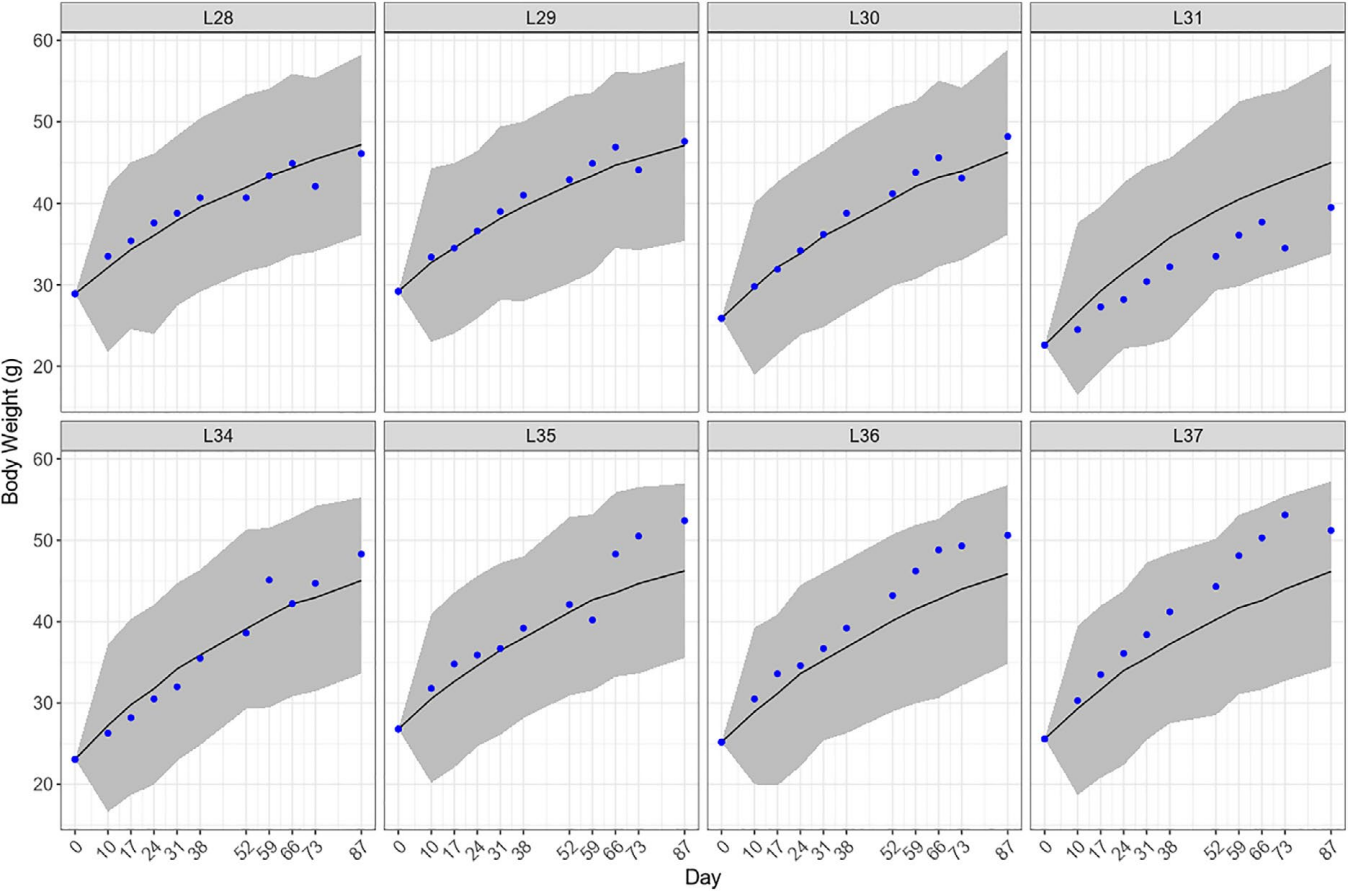
Body weight predictions for E3L.CETP mice in the Rozendaal study. Each panel represents a mouse. The solid line refers to the mean of predictions. The shade area represents the 95% credible interval of predictions. The blue dots represent the experimental data

Based on our ODE‐based dynamic model, E3L.CETP mice fed by the HFHC diet had a food intake of 16.5 [15.9, 17.0] kcal/day (posterior mean and 95% credible interval), with maximum feed efficiency of 23.3% [21.6%, 25.1%] and a maximum body weight of 55.6 [54.6, 56.6] g. To validate our ODE model, we predicted the body weight changes in eight HFHC diet feeding E3L.CETP mice were included in a previous study (Rozendaal et al., 2018). based on their initial body weight and cumulative food consumption. We found that in all eight E3L.CETP mice, the actual experimental data were within the 95% credible interval of our predictions (Figure 1).

### 3.2 Associations between feed efficiency and gene expression levels in the liver

**FIGURE 2:**
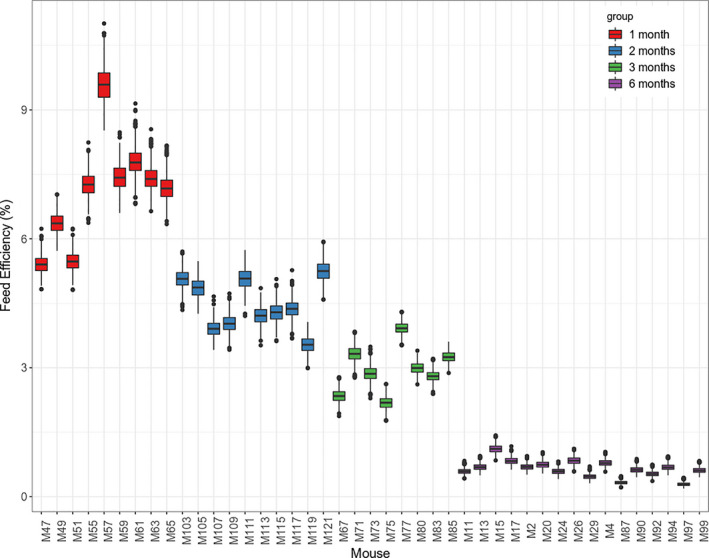
Feed efficiency on the day when the animals were sacrificed. Feed efficiency was calculated based on the dynamic model and the calculation was repeated for 1000 times. Each box represents the results of 1000 simulated feed efficiency values

In order to identify candidate genes and pathways that associated with FE, RNA sequencing was performed on liver tissue derived from 43 E3L.CETP mice. Since these 43 mice were sacrificed on different days and since FE changes over time, we first calculated the FE of these 43 animals (1000 times each) on the day when they were sacrificed based on our dynamic model (Figure 2). The average FE in 1‐month, 2‐month, 3‐month, and 6‐month feeding E3L.CETP mice were 7.11% [6.84%, 7.39%], 4.46% [4.29%, 4.64%], 2.96% [2.75%, 3.16%], and 0.65% [0.53%, 0.79%], respectively.

**FIGURE 3:**
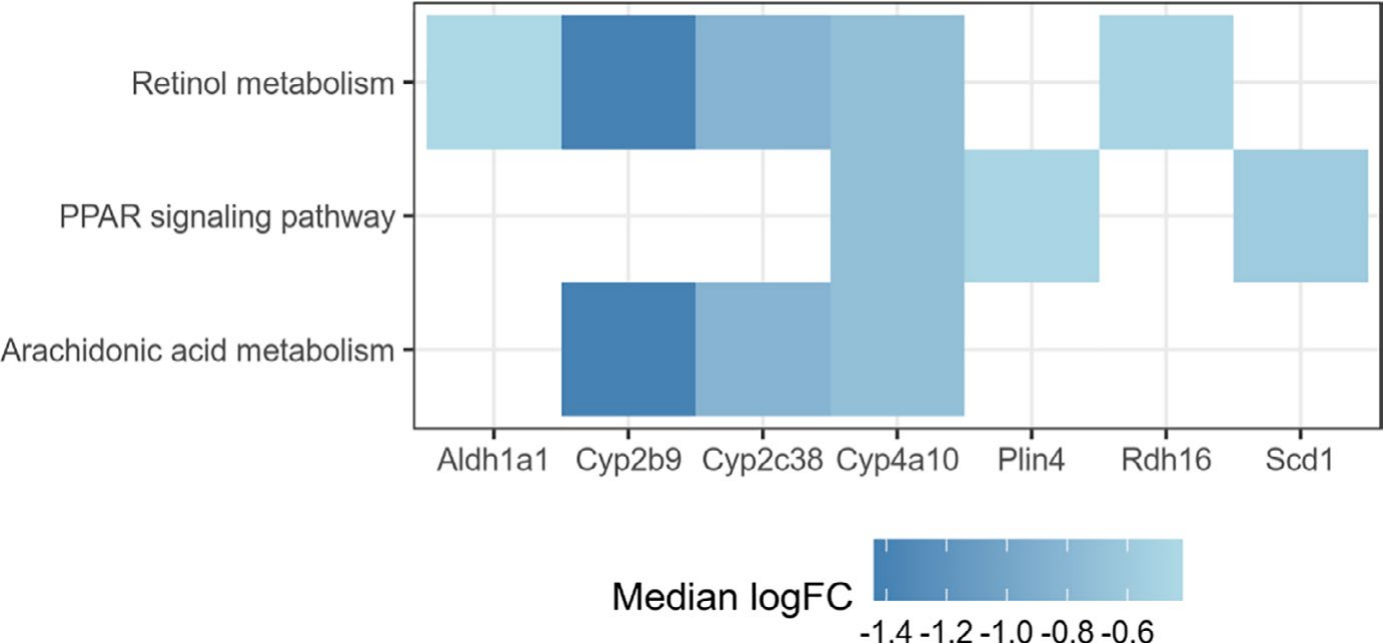
Pathways enriched with down‐regulated genes in the 1‐month feeding group (N = 9). The hypergeometric test was used to calculate the *P* values. The median log fold change was calculated based on 1000 simulations

We observed that higher FE was associated with down‐regulated retinol metabolism pathway in the 1‐month feeding mice group (861 out of 1000 simulations), followed by arachidonic acid metabolism (613 simulations) and PPAR signaling pathway (576 simulations) (Figure 3). In the retinol metabolism pathway, we observed that higher FE was associated with lower expression levels of *Cyp2b9* (median logFC = −1.42, median FDR = 0.00312), *Cyp2c38* (median logFC = −0.834, median FDR = 0.00529), *Rdh16* (median logFC = −0.482, median FDR = 0.00731), *Cyp4a10* (median logFC = −0.706, median FDR = 0.0242), and *Aldh1a1* (median logFC = −0.441, median FDR = 0.0487). *Cyp2b9*, *Cyp2c38*, and *Cyp4a10* also participate in arachidonic acid metabolism. Regarding the PPAR signaling pathway, we observed that higher FE was associated with lower expression levels of *Plin4* (median logFC = −0.475, median FDR = 0.00698), *Scd1* (median logFC = −0.578, median FDR = 0.0192), and *Cyp4a10* (median logFC = −0.706, median FDR = 0.0242).

**FIGURE 4:**
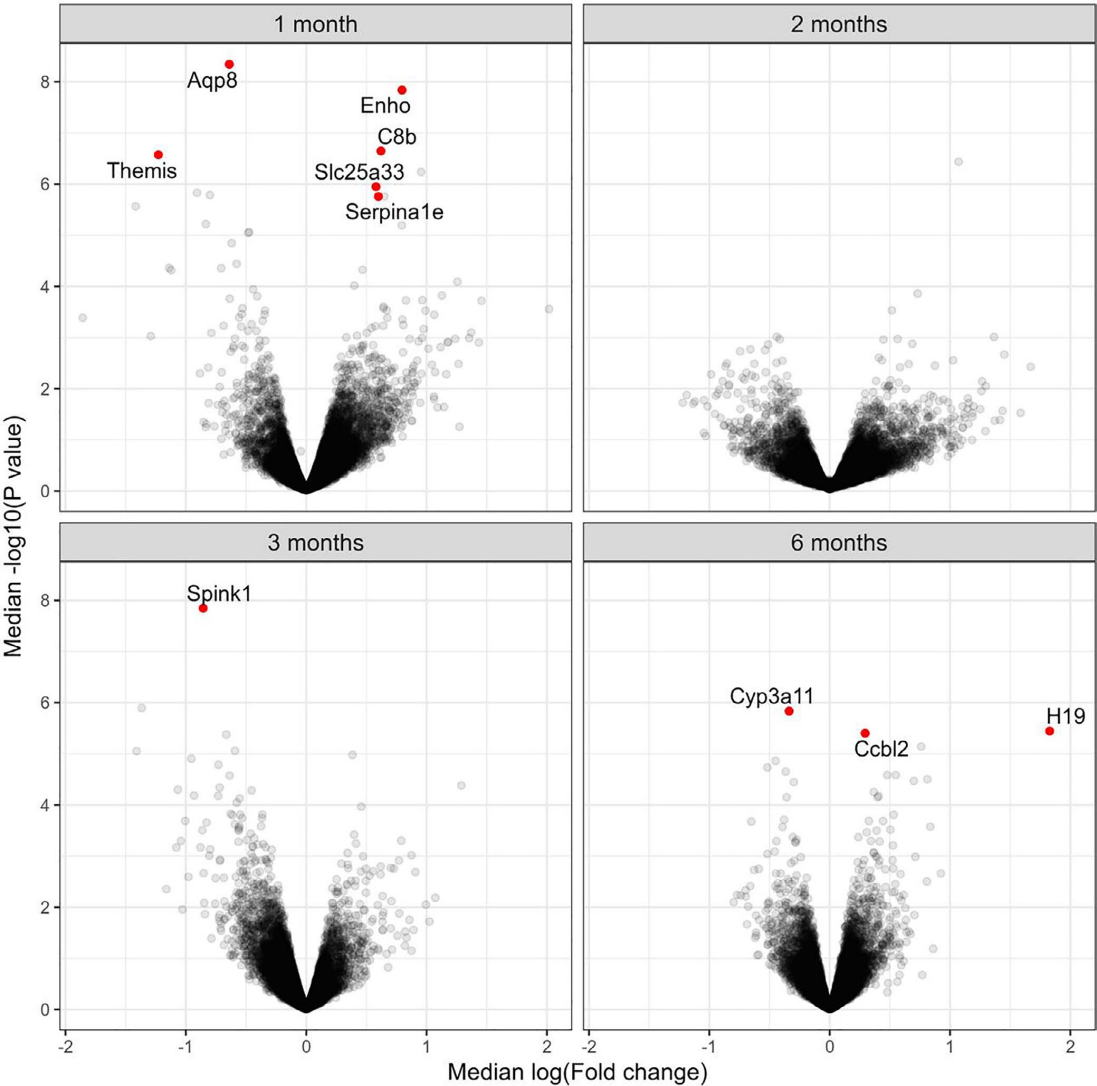
Volcano plots identifying genes that were associated with feed efficiency. Genes with False Discovery Rate below 0.05 (likelihood ratio test) for at least 950 times in 1000 simulations in 1 month (N = 9), 2 month (N = 10), 3 month (N = 8), and 6 month (N = 16) group are presented as dots, respectively

We did not observe any genes or pathways that were associated with FE in the 2‐month feeding groups. In the 3‐month feeding group, we observed that FE was inversely associated with expression levels of *Spink1* (968 simulations, median logFC = −0.856, median FDR = 0.00015). In the 6‐month feeding group, we observed that increasing feed efficiency was associated with increasing expression levels of *Ccbl2* (993 simulations, median logFC = 0.294, median FDR = 0.0154) and *H19* (972 simulations, median logFC = 1.83, median FDR = 0.0136). In contrast, increasing feed efficiency was associated with decreasing expression levels of *Cyp3a11* (999 simulations, median logFC = −0.337, median FDR = 0.00935) (Figure 4).

### 3.3 Relationship between feed efficiency and hepatic triglycerides

**FIGURE 5:**
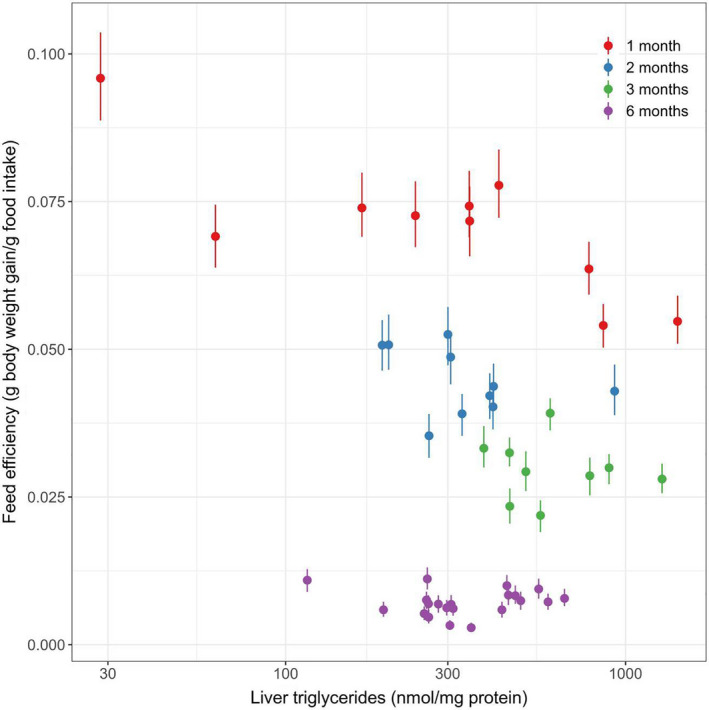
Relationship between feed efficiency and hepatic triglycerides in E3L.CETP mice fed by high‐fat high‐cholesterol diet for 1 month (N = 10), 2 months (N = 10), 3 months (N = 9), and 6 months (N = 21). The short vertical lines represent the 95% credible interval for the feed efficiency

In the 1‐month feeding group, we observed that there was a significant inverse correlation between FE and hepatic triglycerides (Spearman correlation −0.676, 95% credible interval [−0.806, −0.564]) in 726 of 1000 simulations (Figure 5). We did not find a significant correlation between feed efficiency and hepatic triglycerides in 2‐month, 3‐month, and 6‐month feeding group (Figure 5).

### 3.4 Relationship between feed efficiency and hepatic glucose production

**FIGURE 6:**
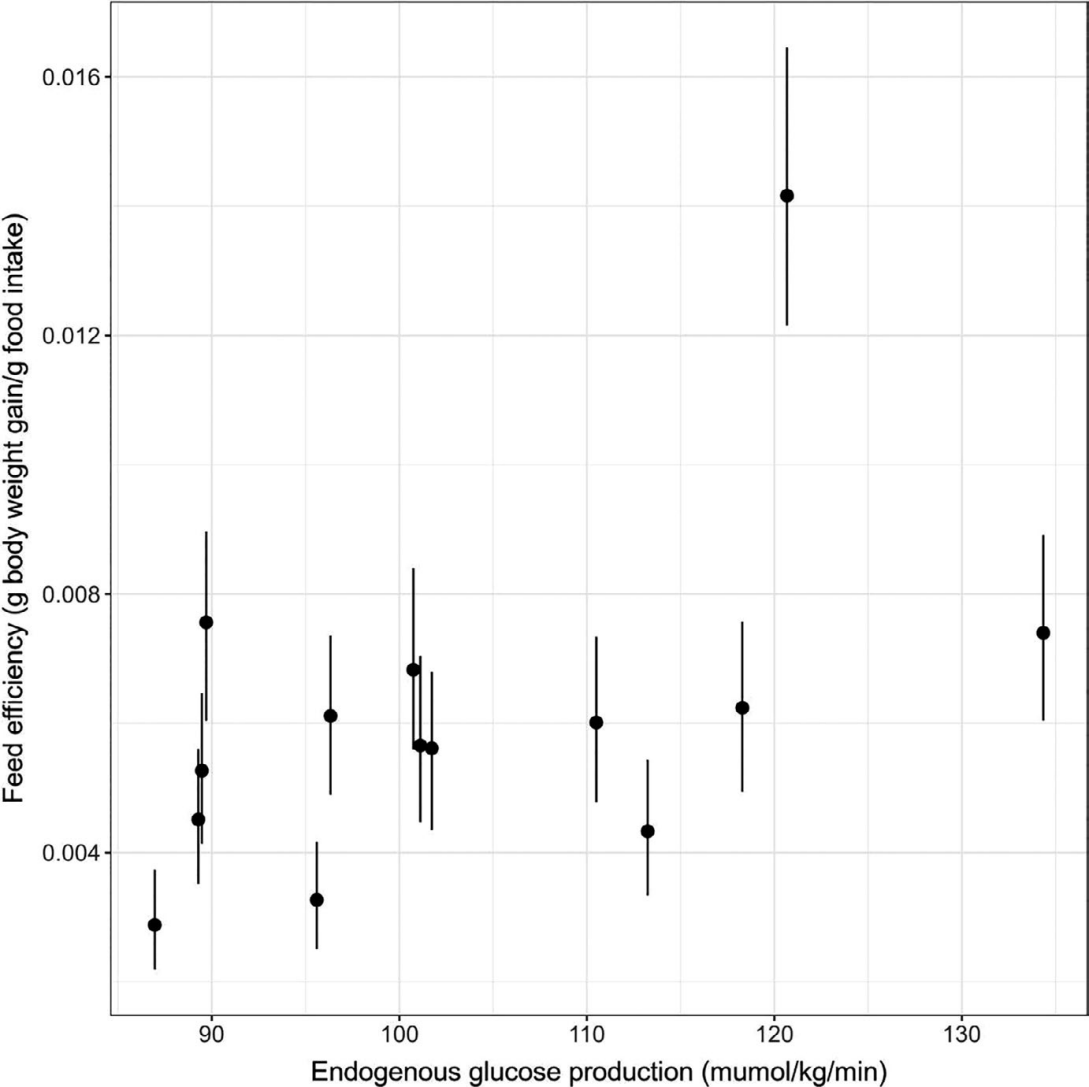
Relationship between feed efficiency and hepatic glucose production in 14 E3L.CETP mice fed by high‐fat high‐cholesterol diet for 6 months. The short vertical lines represent the 95% credible interval for the feed efficiency

In this study, hepatic glucose production was measured in 14 mice from the 6‐month feeding group. We observed that increasing FE was significantly associated with enhanced hepatic glucose production rate (Spearman correlation coefficient 0.550 [0.508, 0.604]) in 854 of 1000 simulations (Figure 6).

## 4 DISCUSSION

Feed efficiency (FE), a ratio monitoring body weight gain per unit of food consumed, is an important economic trait in the livestock industry, in particular, to efficiently increase lean mass by increasing FE. In contrast to maximizing FE in livestock, reducing FE provides an interesting approach to counteract the human obesity pandemic developed over the last decades. As far as we know, biological processes that regulate FE have been sparsely investigated in human studies, most probably due to the difficulty of accurately measuring food intake over prolonged time intervals under constant environmental conditions. Also in this study, we did not study humans but employed a humanized mouse model mimicking the development of metabolic syndrome. In such an animal model it is relatively simple to measure food intake and body weight gain over a prolonged time interval. Our analysis showed that FE in E3L.CETP mice decreased with increasing age. To be able to identify genes associated with FE, it was necessary to develop a computational model calculating FE at time points when gene expression was determined. To this end, we developed an ODE‐based model based on the longitudinal data of food intake and body weight, published in a study on 105 E3L.CETP mice fed a HFHC diet for various periods of time (Paalvast et al., 2017). To validate the model, we had access to the experimental data with the same mice and the same diet in a completely different laboratory setting (Rozendaal et al., 2018). Our ODE‐based model successfully predicted body weight changes in the validation cohort.

Since the liver is generally considered to be the metabolic coordinator of whole body energy metabolism, we studied the relationship between hepatic gene expression and FE.

After feeding HFHC diet for 1 month, we observed that the average FE of E3L.CETP mice was 7% and increasing FE was associated with down‐regulated retinol metabolism. The liver is the most important organ for the storage and metabolism of retinol (vitamin A). It was shown that hepatic vitamin A metabolism regulated FE in pigs (Zhao et al., 2016). We observed that at the 1‐month time point FE was associated with five genes involved in vitamin A metabolism including *Cyp2b9*, *Cyp2c38*, *Rdh16*, *Cyp4a10*, and *Aldh1a1*. Interestingly, in a recent study which also used the E3L.CETP mouse model, it was shown that hepatic expression levels of *Cyp2b9*, *Cyp2c38*, *Rdh16*, and *Cyp4a10* in the mice that developed metabolic syndrome phenotypes with high‐fat diet were different from the ones that did not (Nasias et al., 2019). We found that a 1% increase in FE was associated with a 26% decrease in *Aldh1a1* expression levels. In fact, *Aldh1a1* is a key enzyme in the synthesis of 9‐cis retinoic acid, the potent agonist of both the retinoic acid receptor (RAR) and retinoid X receptor (RXR) (Ziouzenkova & Plutzky, 2008). Deficiency of *Aldh1a1* inhibited 9‐cis retinoic acid synthesis, leading to significantly attenuated hepatic glucose production as well as hepatic triacylglycerol synthesis (Kiefer et al., 2012). Since retinoic acid is also a ligand for the PPAR (Peroxisome Proliferator‐Activated Receptor), inhibited vitamin A metabolism is anticipated to reduce the activity of PPAR signaling pathway (Shaw et al., 2003). Indeed, we found that a 1% increase in FE was associated with downregulation of PPAR signaling pathway genes such as *Plin4*, *Scd1*, and *Cyp4a10* by 28%, 33%, and 39%, respectively. We noticed that both *Plin4* and *Scd1* play important roles in regulating hepatic lipid accumulation (Chen et al., 2013; Li et al., 2009). We also found that a 1% increase in FE corresponded to a 74% increase in *Enho* expression levels. *Enho* (energy homeostasis associated) encodes adropin which governs energy homeostasis. Overexpression of hepatic *Enho* was showed to correlate with decreasing hepatic steatosis (Kumar et al., 2008). This is consistent with our observation that E3L.CETP mice with high FE had low hepatic triglyceride levels.

In the mice fed HFHC diet for 1 month, we also observed that increasing FE was associated with downregulated arachidonic acid metabolism. Arachidonic acid metabolism plays an important role in triggering and resolving inflammation. We noticed that a 1% increase in FE corresponded to downregulation of *Themis* by 57% and upregulation of *Serpina1e* by 52%. *Themis* (thymocyte‐expressed molecule involved in selection) plays an important role in T‐cell development and its deficiency reduced the number of CD8^+^ T cells (important drivers of hepatic inflammation) (Breuer et al., 2020; Lesourne et al., 2009). HFHC diet consumption can cause liver injury (Soltis et al., 2017), stimulating alpha 1‐antitrypsin (encoded by *Serpina1e*) production in response to the inflammation (Janciauskiene et al., 2004). Since fueling the immune response is an energetically expensive process, downregulation of arachidonic acid metabolism and inflammation‐related genes may preserve more energy for growth. Interestingly, a study showed that the hepatic immune response to inflammation indeed affected the FE in cows (Paradis et al., 2015).

Compared to the 1‐month feeding group, the average FE in E3L.CETP mice after 3‐month feeding of HFHC diet decreased to about 3% and its relationship with hepatic gene expression completely changed. We found that a 0.5% increase in FE was associated with a 45% decrease in expression level of *Spink1* (serine protease inhibitor Kazal‐1). *Spink1* promotes cell proliferation and was found upregulated in hepatocellular carcinoma (Marshall et al., 2013). Our observation suggested that the 3‐month feeding E3L.CETP mice may achieve a higher feed efficiency by suppressing hepatocyte proliferation.

After feeding 6 months of HFHC diet, the average FE in E3L.CETP mice was low (0.7%) and the relationship between FE and hepatic gene expression changed again. We found that a 0.1% increase in FE was associated with a 128% increase in expression levels of *H19* and a 11% increase in *Ccbl2*, as well as a 10% decrease in *Cyp3a11*. Here we focused on *H19* because the effect sizes of *Ccbl2* and *Cyp3a11* were small. *H19* encodes a long noncoding RNA (lncRNA) which was found elevated in diet‐induced diabetic mice (Zhang et al., 2018). More interestingly, overexpression of hepatic *H19* was shown to promote hepatic glucose production (Zhang et al., 2018). Our additional analysis showed that 6‐month feeding E3L.CETP mice with high FE tended to have high hepatic glucose production rates. Nevertheless, the feed efficiency difference in the 6‐month was subtle, future studies are required to validate our observations.

Clearly, a limitation of this study is that the genes and pathways identified here were statistically associated with FE. We identified genes that were associated with the feed efficiency mostly in the 1‐month feeding group due to the fact that the feed efficiency showed the greatest variation in this group compared to other feeding groups. In future studies, it would be preferable to determine the association with FE and gene expression in animals divergent in FE. When animals mature FE declines and indeed at 6‐month FE was very low making relations with gene expression less informative. The causal relationship between FE and these genes or pathways is to be investigated in a future study. In this study, we focused on metabolizable energy and did not consider the minor fecal energy losses. Another limitation is that we focused on the relationship between FE and liver physiology. Certainly, it will be important to investigate its relationship with other metabolic organs such as adipose tissues and skeletal muscle, since FE is a complex trait encompassing overall whole‐body metabolism.

## 5 CONCLUSION

We developed a simple ODE‐based model to estimate FE. We applied the model to a cohort of E3L.CETP mice using published data. As expected, FE varied with aging and differed between animals. Our gene expression analysis suggests that various biological processes such as vitamin A metabolism, hepatic response to inflammation and cell proliferation may regulate FE at different stages of diet‐induced obesity.

## CONFLICT OF INTERESTS

The authors declare no competing interests.

## AUTHORS' CONTRIBUTIONS

XZ developed the ODE model, performed the association analyses, and wrote the manuscript. YP and YW performed the animal experiments. PCNR and AKG supervised the project and edited the manuscript. All authors have read and approved the manuscript.
